# Supplementary material for: Disaggregating the evidence linking biodiversity and ecosystem services
Source: Nat Commun. 2016 Oct 7;7:13106. doi: 10.1038/ncomms13106 (PMC5059779; doi:10.1038/ncomms13106)
Supplement: Supplementary Information — Supplementary Figures 1-2, Supplementary Tables 1-3, Supplementary Note 1, Supplementary Methods and Supplementary References [file ncomms13106-s1.pdf]

Supplementary Figures

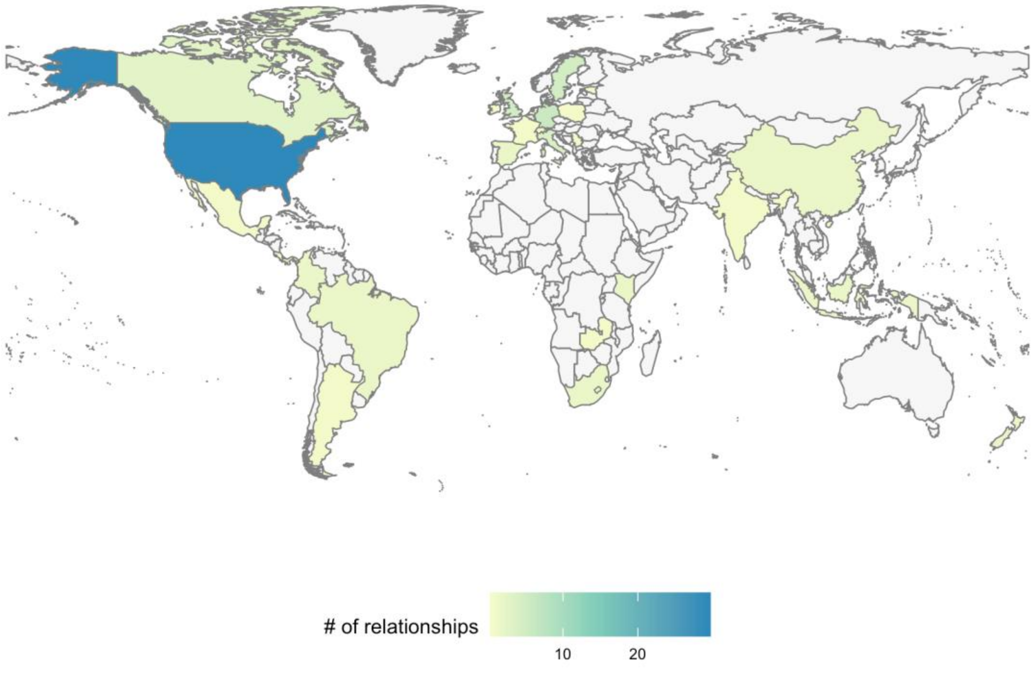

Supplementary Figure 1. Countries in which included studies were conducted. Color gradient depicts the number of relationships for each county: yellow = 0, blue = 30. Regional-scale studies (e.g. across the European Union) are not depicted.

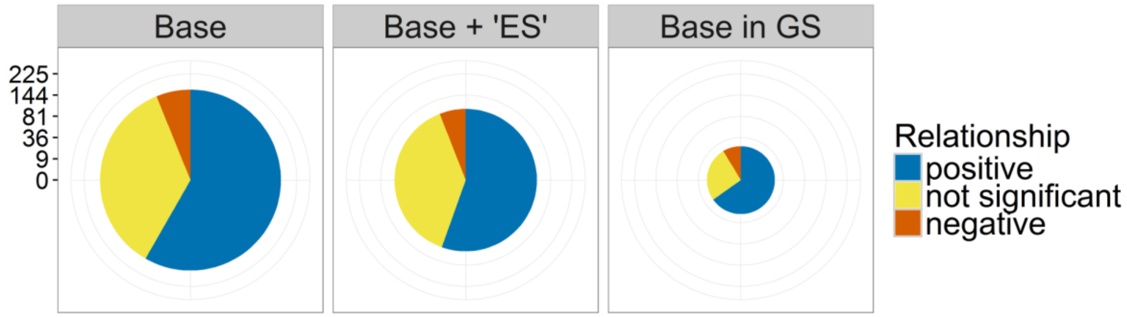

Supplementary Figure 2. Sensitivity analysis for search terms and search engine. Each pie chart depicts the number of relationships and their distribution among positive, negative, and non-significant results. “Base”: original search terms (Supplementary Table 2) and Web of Science. “Base + ‘ES’”: adding ‘ecosystem services’ search term and using Web of Science (comparison to Base: G-test, G = 0.25, df = 2, p = 0.884, n = 264). “Base in GS”: using original search terms and Google Scholar (comparison to Base: G-test, G = 0.92, df = 2, p = 0.632, n = 186). Rightmost two panels depict the subset of papers from our original sample that were also returned by the modified search (see Methods). Colors denote the sign of the reported relationship: blue = positive, red = negative, yellow = not significant.

Supplementary Tables

Supplementary Table 1. Percentage (%) of positive ('pos'), negative ('neg'), and non-significant ('not') relationships. N indicates the number of relationships. These data are used in Fig. 2.

	Carbon storage				Crop pollination				Pest control				Water purification			
	pos	not	neg	N	pos	not	neg	N	pos	not	neg	N	pos	not	neg	N
Total	71	24	5	62	60	35	5	55	37	57	6	54	60	20	20	15
Functional	86	14	0	7	67	33	0	9	53	33	13	15	50	33	17	6
Management	72	24	4	25	56	44	0	16	19	76	4	21	50	0	50	2
Spatial	67	27	7	30	60	30	10	30	44	56	0	18	72	14	14	7

Supplementary Table 2. Search terms used in Web of Science and Google Scholar. These terms follow those in Cardinale et al.[1].

Ecosystem Service	Search term
**Carbon Storage**	(carbon storage OR C-storage) AND (biodiversity OR biological diversity OR diversity)
**Water Purification**	freshwater AND (decontamination OR nutrient OR purification OR quality) AND (biodiversity OR biological diversity OR diversity)
**Crop Pollination**	pollination AND (biodiversity OR biological diversity OR diversity)
**Pest Control**	(biocontrol OR "biological control") AND (agriculture OR agricultural OR crop) AND (pest$ OR prey OR insects OR herbivore$) AND (biodiversity OR biological diversity OR diversity)

Supplementary Table 3. Illustrative measures of ES supply and benefit used for coding papers. We define benefits as positive changes in human well-being, following The Economics of Ecosystems and Biodiversity (TEEB)[2] and Fisher et al.[3]. Benefits need not be measured in monetary terms. If a paper reported both supply and benefit measures, both relationships were coded.

Ecosystem Service	Not included	Supply	Benefit
**Carbon Storage**	Measures of carbon sequestration (which measures rates, not quantities stored)	consider equal to benefit	density of carbon (MT/Ha) stored in aboveground biomass, belowground biomass, or soil
**Water Purification**	Static measures of water quality	Changes in water quality that were not linked to an identified beneficiary, static water quality indices that were clearly identified by authors as proxies for purification service	Increases in water quality that were linked to an identified beneficiary (none found)
**Crop Pollination**	Plant diversity, pollinator abundance	Pollinator visitation rate, pollen deposition	Increased fruit set, fruit weight, seed set, seed weight, fruit quality, retention of developing fruits, seed germination.
**Pest Control**	Predator abundance	Pest survival rates; pest parasitism/ predation rates	Decreased crop damage; increased crop yield

Supplementary Notes

Supplementary Note 1

This note accompanies the methods section to clarify attributes recorded for each relationship and coding decisions for each attribute. Here we do not include methods for paper selection, rules for whether a relationship qualified for inclusion in our dataset, or a description of how relationships were categorized into the three linkage types. That information is presented within the manuscript.

For each relationship included in our dataset, we recorded the following:

1. The linkage type:
    a. Choice between *functional*, *spatial*, and *management*
    b. Functional linkages:
        i. Experimentally manipulate biodiversity and measure the resulting change in ES. No observational studies included in this linkage type.
    c. Spatial linkages:
        i. Measure and compare the response of biodiversity and ES to a spatial gradient in biophysical or anthropogenic drivers of change.
        ii. Typically tested with observational studies.
    d. Management linkages:
        i. Measure and compare the response of biodiversity and ES to a management intervention.
        ii. A management intervention is a discrete choice between options or levels of management (e.g. organic or not, hedgerow or not).
        iii. Can be experimental (i.e., management action is manipulated) or observational (i.e., compare existing differences).
        iv. Gradients of land use do not qualify as management. We considered these to be spatial linkages, just like an elevation, moisture, age, or any other spatial gradient.

2. The country where the study took place.

3. Study design:
    a. Choice between: *observational* or *experimental*
    b. Distinguished by whether the independent variable(s) observed or manipulated.

4. For management relationships, what the management action was.
    a. For example: planting a pollinator enhancement

5. The ES in the relationship:
    a. Choice between: *crop pollination*, *pest control*, *carbon storage*, and *water purification*.

6. The ES measure:
    a. For example, *above ground biomass* or *soil carbon* for carbon storage, *fruit set* or *pollen deposition* for pollination.
    b. The actual attribute reported to measure the ES in question.

7. The units of the ES measure.
    a. For example: *Mt/h, pollinator visits/*m^2*/10minutes*.

8. ES supply or benefit:
    a. Choice between: *supply* or *benefit.*
    b. Distinguishes measures of biophysical supply of the ES from actual benefits to people. See examples of supply and benefit measures in Supplementary Table 3.
    c. If both supply and benefit measures were provided, we recorded them as separate relationships.

9. ES benefit measures:
    a. Choice between: *biophysical, monetary,* or *other* (e.g. health outcomes, number of people effected).

10. The taxonomic identity of biodiversity as listed in the paper:
    a. For example: *birds*, *macroinvertebrates.*
    b. If multiple taxa were related to the same ES measure, we coded a separate relationship for each taxon.
    c. If ES was related to diversity of multiple taxa in a causal chain (i.e., taxon 1 -> taxon 2 -> ES), we coded the relationship involving the most proximate taxon for which biodiversity (instead of abundance) was measured. For instance:
        1. Plant diversity → predator diversity → ES;
           code: predator diversity → ES
        2. Plant diversity → predator abundance → ES
           code: plant diversity → ES
        3. Natural ecosystem abundance → predator abundance → ES
           code: predator abundance → ES, but record the diversity measure as “abundance” (see section 12).

11. Taxonomic resolution of diversity:
    a. Choice between *genetic, functional, species, taxonomic* (genus/family/some taxonomic group larger than species), or *ecosystem* (e.g. land cover types, forest types)
    b. Where the same taxon was measured at multiple resolutions (e.g. relating both species-level and functional diversity of bees to crop production), we coded these as separate relationships.

12. The diversity measure:
    a. Choice between: *richness*, *index*, *abundance*, or *multiple.*
    b. Because abundance is not a true measure of diversity, we excluded all relationships involving abundance from analyses.
    c. If both abundance and diversity were related to a ES, we only recorded the diversity relationship.
    d. If biodiversity of the same taxon, at the same resolution, was measured in multiple ways (e.g. species richness and species diversity index), we collapsed this into a single relationship:
        i. If the paper indicated a preference between these measures, we coded the preferred measure.
        ii. If the paper did not indicate a preference, we reported the sign of the majority of reported relationships.

13. Whether the biodiversity measure in the relationship is for a service provider of the ES:
    a. Choice between *yes* and *no*.
    b. There was one exception where we recorded "yes" in this column even if the coded biodiversity is not the service provider. If the service provider was one element of a causal chain linking the ES to several taxa (see section 10c above), we recorded this as *yes*.
    c. If biodiversity was reported for a group of taxa, of which only one was the service provider (e.g., relating pollination ES to an aggregate index of bird, bee, butterfly, and mammal diversity), we recorded *no*.

14. The condition or treatment for the relationship:
    a. For example: *high vs. low fertilizer treatments, stable and variable stream velocities*.
    b. If the relationship between the same unit of biodiversity and the same ES was reported under multiple conditions, we either reported separate relationships or collapsed into a single relationship according to the following rules:

        i. Where relationships are tested under different treatments (i.e., relating diversity and carbon storage under high and low fertilizer treatments):
            1. If the author presented a pooled result across treatments, we reported the most general reported relationship.
            2. If the author did not report a pooled result, we reported each treatment as a separate relationship.
        ii. For carbon storage studies:
            1. We coded different pools of carbon (e.g., above ground, below ground, soil) as separate relationships
            2. For soil carbon, we pooled soil depths if authors reported them separately according to a majority rule: if a majority of depths were positive (or negative), we coded as positive (or negative). If equal numbers were positive and negative, we coded as non-significant

15. Extent:
    a. For example: *150,000* m^2
    b. For field studies, we used the total area needed to encompass all of the sample sites (e.g., a study with 15 1-ha farms scattered across the state of Vermont has an extent of Vermont, not 15 ha).
    c. For lab experiments, we assumed extent was equal to grain*sample size.

16. Grain:
    a. For example: $5m^2$
    b. The spatial scale of the unit of analysis at which ES and biodiversity measures were compared (but not subsamples of those units).
    c. For instance: if in each of 15 fields, an author measured fruits produced on 10 bushes, assessed pollinator diversity along five 10 m transects, and then calculated a pooled or mean value for each variable in each field, we considered each field to be a data point representing aggregated diversity and ES measures. We coded grain as the field, not the transect or the bush.

17. The sign of the relationship:
    a. Choice between *positive*, *negative*, and *non-significant*.
    b. Positive if:
        i. An increase in biodiversity resulted in an increase in ES
        ii. ES and biodiversity were both positively or both negatively correlated to a management action or spatial gradient
    c. Negative if:
        i. An increase in biodiversity resulted in a decrease in ES

    ii. ES and biodiversity were oppositely and significantly correlated to a management action or spatial gradient (one positive and one negative)

  d. Non-significant if:
    i. A change in biodiversity had a non-significant impact on ES
    ii. Either ES or biodiversity was non-significantly correlated to a management action or spatial gradient.
    iii. ES and biodiversity were both non-significantly correlated to a management action or spatial gradient.

18. Whether the relationship reported in the paper or inferred by the coder:
  a. Choice between *reported* and *inferred*:
    i. Reported: if the paper directly reported on the relationship between biodiversity and ES.
    ii. Inferred: if the paper reported biodiversity and ES relationships separately, and the sign of the relationship between them was inferred based on whether they showed the same response to a third variable or not.

19. Whether a non-linear relationship was tested:
  a. Choice between *yes* and *no*.
  b. *Yes* if the paper clearly stated that both linear and nonlinear relationships were tested, and the best fit was selected.

20. Whether a non-linear relationship was reported:
  a. Choice between *linear* and *non-linear*.
  b. Recorded regardless of whether the paper tested both and selected the best fit.

Supplementary Methods

We tested the sensitivity of our results to the search terms and search engine used to extract papers for review. To investigate potential search terms bias, we tested the effect of adding “ecosystem services” to our search term list. Using Web of Science, we found 27% of our original papers in the top-ranked 563 papers resulting from the new search. This subset of papers showed an increased proportion of papers with relationships and an increased number of relationships per paper, compared to our original complete sample. However, we found no significant differences in proportions of positive, negative and non-significant relationships between this subset and our complete set of relationships (Supplementary Fig. 2). The low degree of overlap between searchers, however, illustrates that any literature search is sensitive to search terms used. We elected to use our original search terms for consistency with earlier work[18] and to capture relevant papers that do not use the specific phrase ‘ecosystem services’.

To investigate sensitivity to search engine, we repeated our literature search using the same search terms in Google Scholar, rather than Web of Science. We found 8% of our original papers in the top-ranked 563 papers resulting from the new search. Using Google Scholar decreased the proportion of papers with relationships, and increased the number of relationships per paper; however, this subset of papers did not yield significantly different relationships to those reported in our paper (although the subset sample size for comparison was small) (Supplementary Fig. 2). The two search engines employ differing (and proprietary) algorithms for ranking papers. Rankings in Google Scholar, for example, depend in part on the number of times a paper has been cited, while rankings for Web of Science focus largely on keywords and text. We elected to use the Web of Science results, to again remain consistent with earlier reviews[18] and to capture the relevant published evidence regardless of subsequent citation rates.

These are partial sensitivity tests, in that they compare our full set of papers to a subset of those papers also returned by the modified searches. A full sensitivity test would require coding all additional papers found when adding a search term or using an alternative search engine.

Because our primary objective was not to compare search terms and search engines, we did not perform this additional task.

Supplementary References

1. Cardinale, B. J. *et al.* Biodiversity loss and its impact on humanity. *Nature* **486,** 59–67 (2012).
2. TEEB. The economics of ecosystems and biodiversity: Ecological and economic foundations (Earthscan, 2010). doi:10.1017/s1355770x11000088
3. Fisher, B., Turner, R. K. & Morling, P. Defining and classifying ecosystem services for decision making. *Ecol. Econ.* **68,** 643–653 (2009).
